# Impact of positioning errors in the dosimetry of VMAT left-sided post mastectomy irradiation

**DOI:** 10.1186/s13014-020-01556-w

**Published:** 2020-05-07

**Authors:** Xiongfei Liao, Fan Wu, Junxiang Wu, Qian Peng, Xinghong Yao, Shengwei Kang, Yanqun Zhao, Lucia Clara Orlandini

**Affiliations:** Sichuan Cancer Hospital & Research Institute, School of Medicine University of Electronic Science and Technology of China, 55th Renmin South Road, 4th Section, Chengdu, 610041 China

**Keywords:** Radiotherapy, Breast cancer, Volumetric modulated arc therapy, Positioning errors, Heart dose

## Abstract

**Background:**

Volumetric modulated arc therapy (VMAT) adopted in post-mastectomy radiation therapy (PMRT) has the capacity to achieve highly conformal dose distributions. The research aims to evaluate the impact of positioning errors in the dosimetry of VMAT left-sided PMRT.

**Methods:**

A total of 18 perturbations where introduced in 11 VMAT treatment plans that shifted the isocenter from its reference position of 3, 5, 10 mm in six directions. The thoracic wall and supraclavicular clinical target volumes (CTVs), the heart and the left lung dose volume histograms (DVHs) of 198 perturbed plans were calculated. The absolute differences (*∆*) of the mean dose (Dm) and DVH endpoints Vx and Dy (percentage volume receiving x Gy, and dose covering y% of the volume, respectively) were used to compare the dosimetry of the reference vs perturbed plans.

**Results:**

Isocenter shifts in the anterior and lateral directions lead to maximum disagreement between the CTVs dosimetry of perturbed vs reference plans. Isocenter shifts of 10 mm shown a decrease of D95, D98 and Dm of 12.8, 18.0, and 2.9% respectively, for the CTVs. For 5 mm isocenter shifts, these differences decreased to 3.2, 5.2, and 0.9%, respectively, and for 3 mm shifts to 1.0, 1.7, and 0.6%, respectively. For the organs at risk (OARs), only isocenter shifts in the right, posterior and inferior directions worsen the plan dosimetry, nevertheless not negligible lung *∆* V20 of + 2.6%, and heart *∆* V25 of + 1.6% persist for 3 mm shifts.

**Conclusions:**

Inaccuracy in isocenter positioning for VMAT left-sided PMRT irradiation may impact the dosimetry of the CTVs and OARs to a different extent, depending on the directions and magnitude of the perturbation. The acquired information could be useful for planning strategies to guarantee the accuracy of the treatment delivered.

## Background

Post-mastectomy radiation therapy (PMRT) is technically difficult, given the complexity of the target volume and its proximity to critical structures, including the heart, lung, brachial plexus, and contralateral breast [1–3]. More advanced techniques, such as intensity modulated radiation therapy or volumetric modulated arc therapy (VMAT) can achieve highly conformal dose distributions with improved target volume coverage and sparing of normal tissues compared to conventional techniques [4]. These techniques have the potential to improve treatment outcomes for PMRT and significantly reduce the dose to the heart and the ipsilateral lung [5–8]. Nevertheless, uncertainties related to inter-fraction positioning may lead to inaccuracies in the dose delivered [9]; the steepness of the dose-effect curves can limit the efficacy of VMAT, thus affecting patient outcomes for both local tumor control and normal tissue complications. The radiation dose delivered to the heart should be monitored as even lower doses of radiation to the heart may lead to a relevant injury [10, 11]. As previously reported [12–15], dose differences of breast treatment in the supine position can be correlated with the patient setup. Image guidance is frequently used to detect large setup errors and refine patient positioning in the setting of radiotherapy [16]. Modalities such as electronic portal image devices, cone beam computed tomography, and surface imaging have been implemented to verify setup accuracy. However, daily shifts in patient setup are inevitable even with image guidance [17].

To buffer these daily setup errors and ensure adequate dosing to target tissue, the planning treatment volume incorporates a margin to the clinical target volume taking into consideration the systematic and random errors of daily setup [18]. This study’s aim is to evaluate the impact of positioning errors on the dosimetry of postmastectomy VMAT irradiations that are specifically caused by isocenter positioning variations. The study was conducted using treatment plans delivered in the clinical practice and provides details on the isocenter direction misalignment, that contribute to under/overdosage of the targets/organs at risk (OARs). The acquired information could be useful for planning strategies and optimization to improve overall treatment accuracy.

## Methods

This retrospective study included the analysis of 198 perturbed treatment plans and corresponding dose volume histograms (DVHs) of 11 cases of left-sided breast cancer patients who underwent PMRT. The patients were immobilized in the supine position using a WingSTEP breast board with head holder and a KneeSTEP knee support (Elekta, Stockholm, Sweden). A sheet of water-equivalent bolus (*ρ* = 1 g·cm − 3) with dimensions of 28 × 28 × 1 cm3, placed over the skin in correspondence with the treatment field was used during the planning computed tomography (CT) and for all treatments; as many institutions reported [19] the chest bolus was used to enhance the radiation dose for the chest-wall surface and to decrease the risk of local recurrence. CT scans with a slice thickness of 3 mm, acquired with a Philips Big bore (Philips, Eindhoven, The Netherlands), were imported into the Pinnacle 3TM Version 9.10 (Philips Medical Systems, Eindhoven, the Netherlands) treatment planning system. A 6 MV photon beam of Axesse linac (Elekta, Stockholm, Sweden), mounting a 160 multileaf collimator Agility (Elekta, Stockholm, Sweden) with a leaf width of 5 mm at the machine isocenter over the full field of 400 mm × 400 mm, available in the department, was used for the treatments.

Experienced radiation oncologists contoured the target and OARs according to the breast cancer atlas for the radiation therapy planning consensus definitions of the Radiation Therapy Oncology Group [20, 21]. Two different clinical target volumes were delineated contiguously: the thoracic wall (CTVth) and the supraclavicular region (CTVsv). The planning target volume (PTV) was obtained with a 3 mm isotropic expansion of the CTVs in all directions except in the adjacent borders of the contiguous targets. The treatments were performed using VMAT with two 200° opposite partial arcs with a start angles between 300° – 340°, and stopping angles between 140° – 180°, and with a collimator rotation of 10°–15°. The variability of 40° in the start/stop gantry angles and of 5° of the collimator rotation’s, was due to the patient treatment plan’s own anatomy. The prescription dose was 50 Gy in 25 fractions. The treatment plan was optimized with the following criteria: at least 95% of the PTV volume receiving 50 Gy and 95% of the prescription dose (V95%) covering at least 99% of the PTV volume; the hot spot defined as PTV receiving more than 110% of the prescription dose as little as possible; less than 20% of the left lung to 20 Gy (V20); less than 10% of the heart to 30 Gy (V30); and a minimized dose to the contralateral lung and breast because some of the beams could penetrate the patient’s right lung and right breast.

For each of the 11 cases, 18 perturbations were introduced in the treatment plans that shifted the isocenter from its reference position of 3, 5, 10 mm in the inferior, superior, anterior, posterior, right and left directions with respect to the patient coordinates. The corresponding perturbed plan was recalculated on the planning CT without varying any parameter other than the isocenter position. A total of 198 perturbed plans were analysed and compared with the corresponding reference ones. The investigated perturbed plans were obtained for specific set-up shifts and not for the complete treatment.

The DVHs of the target’s, left lung and heart were obtained for each perturbation introduced and case studied. The absolute difference of the mean dose (Dm) and of the dose volume histogram endpoints Vx and Dy (percentage volume receiving x Gy, and dose covering y% of the volume, respectively), were used to compare the dosimetry of reference versus the perturbed plans; particularly *∆* Dm, *∆* V25, and *∆* V40 for the heart; *∆* Dm, *∆* V20, and *∆* V40 for the left lung; and *∆* D95, *∆* D98, *∆* Dm, and *∆* D1 for both targets (CTVth and CTVsv).

## Results

Table 1 presents the dosimetric parameters of the reference treatment plans approved and clinically delivered for the 11 cases studied. Tables 2 and 3 presents respectively, the mean absolute differences and the standard deviation of the targets and OARs DVH dosimetric parameters obtained between the perturbed and the corresponding reference plans for each isocenter shift. The absolute differences were given for the dosimetric parameters obtained with the perturbed plan minus those obtained with the reference plan.

**Table 1:** Dosimetry of the reference plans for the cases studied

Patient #	1	2	3	4	5	6	7	8	9	10	11	Mean
**CTVth**^a^												
Vol^b^	221.2	475.6	203.4	158.6	219.4	229.1	308.7	188.4	194.5	155.9	204.9	230.4
D95^c^	50.0	49.7	50.2	50.3	50.4	50.1	50.9	50.1	50.0	50.0	49.9	50.1
D98^c^	49.2	49.3	49.0	49.9	49.8	49.3	49.1	49.3	49.4	49.1	49.2	49.3
D1cc^d^	54.2	53.9	54.8	53.6	53.8	53.6	53.9	53.5	54.0	54.1	54.7	54.1
Dm ^e^	51.7	51.5	51.3	51.9	51.6	52.1	52.1	51.9	52.2	51.9	51.7	51.8
**CTVsv**^a^												
Vol^b^	303.2	258.0	160.3	153.5	153.1	132.5	301.4	195.5	178.8	168.7	178.3	196.8
D95^c^	50.6	49.3	50.0	50.3	50.0	51.1	50.4	50.0	50.7	50.0	50.1	50.2
D98 ^c^	50.0	49.0	49.0	49.9	48.9	49.9	49.9	49.9	50.3	49.1	49.6	49.6
D1cc^d^	54.6	53.1	54.4	53.9	53.1	54.9	54.1	53.4	53.9	52.9	53.0	53.7
Dm^f^	51.7	51.4	51.8	51.7	51.6	51.9	52.0	51.6	52.3	51.2	51.2	51.6
**Heart**												
V25^f^	4.5	4.0	2.0	2.0	1.0	0.5	5.0	1.0	1.0	2.0	1.5	2.2
V40 ^f^	2.0	0.0	0	1.1	0	0	2	0.0	0	0	0	0.4
Dm ^e^	7.5	6.5	9.2	7.3	6.1	6.2	9.2	7.1	6.9	5.3	5.9	6.9
**Left Lung**												
V20 ^f^	20.0	21.0	17.0	15	14.0	15.0	17.0	14.3	18.1	17	16.8	16.9
V40 ^f^	8.0	7.5	5.5	3.0	2.0	2.0	8.0	3.4	8.2	4.2	3.7	4.9
Dm^e^	11.9	12.7	11.2	9.9	9.1	9.7	11.0	9.5	10.8	10.9	9.6	10.5

CTVth^a^, CTVsv^a^: thoracic wall and supraclavicular clinical target volume, respectively; Vol^b^: volume expressed in cm^3^; Dy^c^: dose expressed in Gy covering y% of the volume; D1cc^d^: dose expressed in Gy covering 1 cm^3^ of the volume; Dm^e^: mean dose expressed in Gy; Vx^f^: volume expressed in % receiving a dose of x Gy

**Table 2:** Mean absolute difference and standard deviation of the CTVs DVH dosimetric parameters in the 11 cases studied

Perturbation		CTVth ^a^				CTVsv ^a^			
Shift	Direction	*∆*D95^b^	*∆*D98^b^	*∆*D1^c^	*∆*Dm^d^	*∆*D95^b^	*∆*D98^b^	*∆*D1^c^	*∆*Dm^d^
3 mm	inferior	−0.4 ± 0.4	− 0.5 ± 0.6	0.0 ± 0.1	0.0 ± 0.1	− 0.4 ± 0.6	− 0.5 ± 0.4	0.0 ± 0.1	− 0.4 ± 0.4
	superior	− 0.4 ± 0.5	− 0.5 ± 0.4	0.0 ± 0.1	− 0.2 ± 0.1	− 0.3 ± 0.6	− 0.7 ± 0.5	0.2 ± 0.1	− 0.5 ± 0.6
	posterior	− 0.1 ± 0.3	− 0.3 ± 0.3	0.0 ± 0.0	− 0.2 ± 0.1	−0.1 ± 0.6	−0.5 ± 0.2	0.2 ± 0.1	−0.3 ± 0.3
	right	−0.3 ± 0.2	−0.5 ± 0.2	0.2 ± 0.2	−0.4 ± 0.3	−0.3 ± 0.1	−0.5 ± 0.3	0.3 ± 0.6	−0.4 ± 0.3
	**left**	**− 1.1 ± 0.7**	**−1.5 ± 0.9**	0.4 ± 0.2	0.1 ± 0.2	− 0.5 ± 0.2	−0.6 ± 0.6	0.3 ± 0.4	−0.3 ± 0.1
	**anterior**	**− 1.4 ± 0.9**	**−2.8 ± 1.8**	0.2 ± 0.3	− 0.2 ± 0.2	−0.7 ± 0.6	**− 1.4 ± 0.6**	0.4 ± 0.2	− 0.5 ± 0.6
5 mm	inferior	−1.0 ± 0.3	−1.5 ± 0.7	0.1 ± 0.2	0.0 ± 0.1	−1.6 ± 0.6	− 2.3 ± 1.2	0.3 ± 0.1	− 0.7 ± 0.4
	superior	−1.8 ± 0.4	− 3.2 ± 0.9	0.1 ± 0.2	−0.4 ± 0.2	− 1.1 ± 0.9	−1.8 ± 1.0	0.2 ± 0.6	− 0.7 ± 0.6
	posterior	−0.9 ± 0.4	− 1.8 ± 0.6	0.1 ± 0.2	− 0.4 ± 0.3	−0.7 ± 0.3	−0.7 ± 0.5	0.4 ± 0.3	−0.6 ± 0.4
	right	−1.2 ± 0.7	−1.5 ± 0.7	0.2 ± 0.5	−0.6 ± 0.3	−0.7 ± 0.4	−0.8 ± 0.7	0.5 ± 0.3	− 0.6 ± 0.5
	**left**	**−2.8 ± 1.1**	**−4.3 ± 0.9**	0.8 ± 0.1	0.0 ± 0.3	**− 1.2 ± 0.6**	**−2.3 ± 1.0**	0.6 ± 0.3	− 0.4 ± 0.2
	**anterior**	**− 3.8 ± 1.8**	**−5.7 ± 2.5**	0.5 ± 0.3	− 0.6 ± 0.4	**−2.4 ± 1.0**	**−5.3 ± 1.1**	0.6 ± 0.4	− 0.7 ± 0.6
10 mm	inferior	− 1.7 ± 0.5	−2.2 ± 0.9	0.3 ± 0.3	− 0.8 ± 0.2	- 5.1 ± 2.3	− 8.5 + 2.2	0.4 ± 0.2	− 1.4 ± 0.7
	superior	−6.6 ± 3.4	−8-8 ± 3.1	0.2 ± 0.3	− 1.3 ± 0.7	− 4.1 ± 2.9	− 5.9 ± 3.2	0.3 ± 0.1	− 1.4 ± 0.3
	posterior	−4.9 ± 2.9	−6.6 ± 2.9	0.4 ± 0.7	− 1.5 ± 0.9	−2.7 ± 1.4	−4.5 ± 1.5	0.6 ± 0.2	− 0.8 ± 0.4
	right	−4.6 ± 2.0	−5.4 ± 2.6	0.2 ± 0.6	− 1.8 ± 0.5	−2.5 ± 0.9	−4.7 ± 0.8	0.7 ± 0.1	− 0.8 ± 0.4
	**left**	**− 11.9 ± 5.2**	**−16.1 ± 4.9**	**1.7 ± 0.3**	**−2.5 ± 1.1**	**− 8.3 ± 2.1**	**− 11.6 ± 2.1**	**1.3 ± 0.4**	− 0.8 ± 0.3
	**anterior**	**− 13.6 ± 4.5**	**−17.6 ± 3.6**	**1.0 ± 0.3**	**−3.2 ± 1.8**	**− 11.1 ± 3.4**	**−15.0 ± 4.5**	**1.0 ± 0.6**	**−1.1 ± 0.4**

CTVth^a^, CTVsv^a^: thoracic wall and supraclavicular clinical target volume, respectively; *∆* Dy^b^: absolute difference expressed in Gy of the dose covering y% of the volume; *∆* D1^c^: absolute difference expressed in Gy of the dose covering 1 cm^3^ of the volume; *∆* Dm^d^ absolute difference expressed in Gy of the mean dose. The absolute difference is obtained by subtracting the dosimetric reference value from the corresponding perturbed one. Values in light grey correspond to absolute difference ≤ 1%; in bold isocenter shift directions contributing the most to worsen the target dosimetry and corresponding clinically unacceptable absolute differences

**Table 3:** Mean absolute difference and standard deviation of the OARs DVH dosimetric parameters in the 11 cases studied

Perturbation		Left Lung			Heart		
Shift	Direction	^a^*∆* V20	^a^*∆* V40	^b^*∆* Dm	^a^*∆* V25	^a^*∆* V40	^b^*∆* Dm
3 mm	**inferior**	**1.9 ± 0.6**	**1.6 ± 0.5**	**1.0 ± 0.1**	0.2 ± 0.2	0.1 ± 0.1	0.0 ± 0.1
	superior	− 2.5 ± 0.5	− 1.2 ± 0.7	− 0.8 ± 0.1	− 0.2 ± 0.1	− 0.1 ± 0.1	− 0.2 ± 0.2
	**posterior**	**2.4 ± 0.5**	**2.5 ± 0.5**	**1.1 ± 0.1**	**1.6 ± 1.1**	0.4 ± 0.2	**0.8 ± 0.2**
	**right**	**2.6 ± 0.5**	**2.3 ± 0.7**	**1.2 ± 0.4**	0.6 ± 0.2	0.3 ± 0.3	0.2 ± 0.2
	left	− 2.9 ± 0.8	− 1.9 ± 0.8	−1.0 ± 0.3	− 0.5 ± 0.1	− 0.2 ± 0.2	− 0.4 ± 0.2
	anterior	− 3.0 ± 0.8	− 1.8 ± 0.3	−1.0 ± 0.1	−1.8 ± 0.5	− 0.2 ± 0.1	− 0.6 ± 0.2
5 mm	**inferior**	**3.5 ± 0.5**	**3.1 ± 0.8**	**1.5 ± 0.2**	**0.6 ± 0.9**	0.3 ± 0.1	0.3 ± 0.2
	superior	− 3.6 ± 0.5	−1.8 ± 0.9	−1.2 ± 0.2	−0.8 ± 0.7	−0.2 ± 0.1	−0.4 ± 0.2
	**posterior**	**4.1 ± 0.6**	**4.2 ± 0.7**	**1.7 ± 0.5**	**3.0 ± 1.2**	**1.3 ± 0.8**	**1.4 ± 0.4**
	**right**	**4.3 ± 1.3**	**4.1 ± 1.3**	**2.0 ± 0.4**	**0.9 ± 1.0**	0.4 ± 0.2	0.3 ± 0.2
	left	− 4.5 ± 1.1	−3.0 ± 1.7	−1.6 ± 0.4	−0.8 ± 0.7	−0.3 ± 0.3	−0.5 ± 0.3
	anterior	−5.6 ± 1.6	− 4.1 ± 2.5	−1.7 ± 0.2	− 2.5 ± 1.3	−0.3 ± 0.2	− 1.0 ± 0.5
10 mm	**inferior**	**7.1 ± 1.4**	**6.5 ± 1.1**	**3.1 ± 0.6**	**1.1 ± 1.0**	0.5 ± 0.3	0.2 ± 0.2
	superior	−6.5 ± 1.5	−2,7 ± 1.1	−2.2 ± 0.4	−1.0 ± 1.0	−0.4 ± 0.2	−0.2 ± 0.1
	**posterior**	**8.4 ± 1.2**	**8.4 ± 1.6**	**3.7 ± 0.5**	**7.0 ± 2.2**	**3.6 ± 2.6**	**3.0 ± 0.9**
	**right**	**9.8 ± 1.8**	**8.5 ± 2.5**	**4.1 ± 0.8**	**2.1 ± 1.2**	0.7 ± 0.4	0.4 ± 0.2
	left	− 8.5 ± 1.8	− 4.13 ± 2.4	−2.8 ± 0.9	−1.4 ± 1.1	−0.4 ± 0.2	−1.1 ± 0.9
	anterior	−9.6 ± 1.8	−4,2 ± 2.4	−3-0 ± 0.4	−3.4 ± 2.6	− 1.1 ± 0.9	−2.3 ± 1.0

^a^*∆* Vx: absolute difference expressed in % of the volume covered by a dose of x Gy; ^b^*∆* Dm absolute difference expressed in Gy of the mean dose. The absolute difference is obtained by subtracting the dosimetric parameter reference value from the corresponding perturbed one. In bold the isocenter shift directions contributing to worsen the OARs dosimetry and clinically unacceptable absolute differences

Significant absolute percentage differences were registered for isocenter shifts of 10 mm with a mean decrease for CTVth *∆* D95, *∆* D98, and *∆* Dm of 14.4% (range 3.4–27.1), 19.1% (range 4.5–35.7), and 3.7% (range 1.5–6.2), respectively; while for CTVsv *∆* D95, *∆* D98, and *∆* Dm mean decreases of 11.2% (range 5.0–22.1), 16.9% (range 9.1–30.2), and 2.1% (range 1.5–2.7), respectively, were registered. The maximum dose for both targets was only slightly affected and only for the anterior and right directions with a maximum increase of 1.7 Gy. For 5 mm isocenter shifts, *∆* D95, *∆* D98, and *∆* Dm decreased to 3.8% (range 1.8–7.6), 6.1% (range 3.0–11.6), and 0.6% (range 0.0–1.2), respectively, for CTVth and to 2.6% (range 1.4–4.8), 4.4% (range 1.4–10.7), and 1.2% (range 0.8–1.4), respectively, for CTVsv. For 3 mm isocenter shifts, lower mean differences were found: *∆* D95, *∆* D98, and *∆* Dm decreased to 1.2% (range 0.2–2.8), 2.0% (range 0.6–5.6), and 0.4% (range 0.0–0.8), respectively, for CTVth, and to 0.8% (range 0.2–1.4), 1.4% (range 1.0–2.8), and 0.8% (range 0.6–1.0), respectively, for CTVsv.

For the OARs, only isocenter shifts in the right, posterior, and inferior directions worsen the plan dosimetry; particularly, for the left lung means *∆* V20, *∆* V40, and *∆* Dm of + 8.4%, 7.8% and 3.6 Gy, + 3.9%, 3.8%, and 1.7 Gy, and + 2.3%, + 2.1%, and + 1.1 Gy, were registered for 10, 5, 3 mm isocenter shifts. For the heart, the higher difference was registered for isocenter shifts in the posterior direction with mean *∆* V25 and *∆* Dm of + 7.0%, and 3.0 Gy, of + 3.0%, and + 1.4 Gy, and of + 1.6% and + 0.8 Gy, respectively, for 10, 5, 3 mm isocenter shifts.

## Discussion

**Fig. 1:**
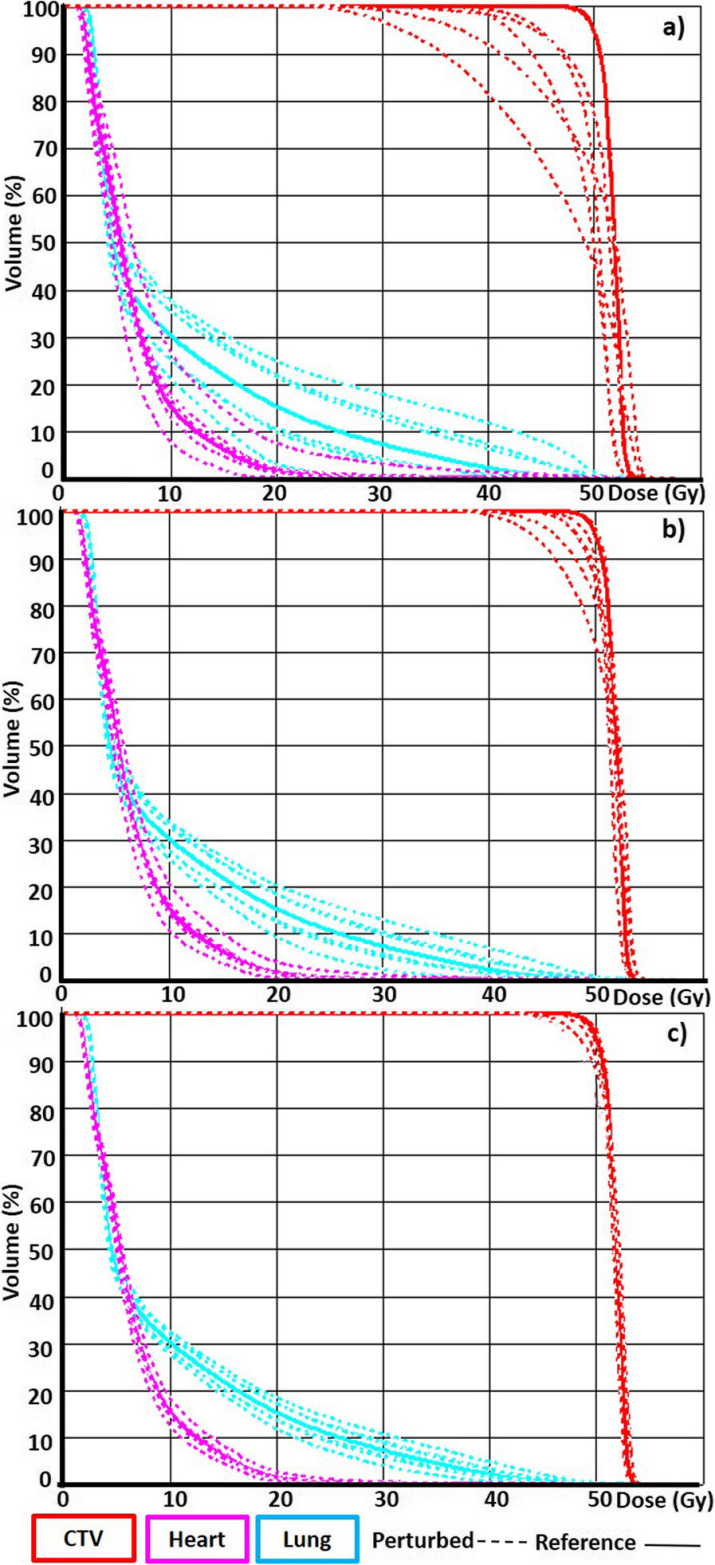
DVHs of the reference and perturbed plans for a representative patient. The DVH of the plans perturbed by isocenter shifts of 10, 5, 3 mm were reported in panel **a**, **b**, **c**, respectively

In this study, the impact of positioning errors on the plan dosimetry of a VMAT PMRT was investigated for three different magnitudes and six different directions of isocenter shifts. The results obtained make it possible to share some considerations. As expected, the impact on the plan dosimetry resulting from a misalignment of the isocenter increases with the magnitude of the isocenter shift, generally for both targets and OARs; nevertheless, shifts of 3 mm that slightly affect the target coverage could be relevant for the increase of dose of the OARs as shows Fig. 1 for a representative patient; the heart, lung, and CTVth DVHs were portrayed for 10, 5, and 3 mm isocenter shifts.

The directions that most affect the coverage of both targets (highlighted in bold in Table 2) are the anterior and left directions. This is understandable because the proximity of the heart and lung to the target in the posterior direction quickly decreases in respect to the target’s coverage when the isocenter shift is in the opposite direction. This is due to the strong dose gradient that must be present between target and heart to ensure an accurate dose coverage of the first without affecting the second; the dose gradient is highly accentuated in cases of modulated intensity treatments. The same applies to the left direction, because the lateral gradient must be steep to avoid as much as possible a dose to the contralateral breast. Isocenter perturbations in the inferior and superior directions have lower impacts on the dose coverage of the CTVth and CTVsv, respectively, because both targets are contiguous, while the opposite directions superior for CTVth and inferior for CTVsv lead to a non-negligible targets underdosage. We can also observe that 3 mm isocenter shifts only affect the dosage of the targets slightly, as expected, considering that the treatment plan was optimized on the PTV obtained with an isotropic expansion of 3 mm around the CTV. These results are aligned with other studies [22] whose authors have found that shifts in the position of the isocenter as large as 3 mm tend to have a modest impact on the quality of VMAT plans.

**Fig. 2:**
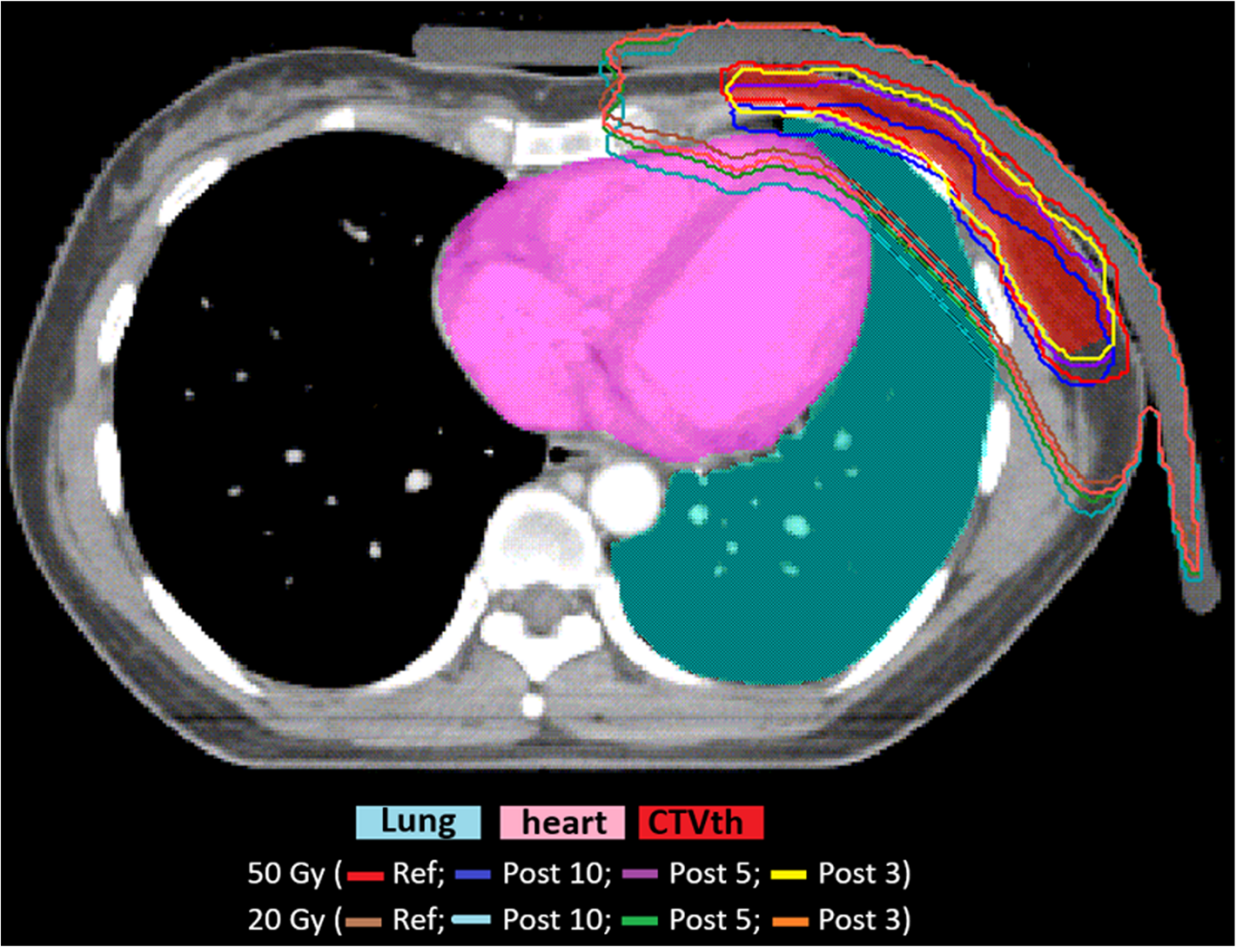
CT transversal scan of a representative patient with isodoses lines of the reference and corresponding perturbed plan. CT transversal slice of a representative patient with 50 Gy, and 20 Gy isodose lines of the reference plan (Ref) and of the corresponding plan perturbed with isocenter shifts in the posterior direction of 10, 5, 3 mm (Post 10, Post 5, Post 3)

The dosimetric disagreement of the OARs must be analyzed closely and at the same time as that of the targets. If, in fact, some directions (superior, left, and anterior) do not affect the organs at risk as they move them away from the treatment field, other directions (inferior, posterior, and right) that only slightly affect the variation in target coverage lead to nonnegligible overdoses of the organs at risk as they significantly increase the portion of the lung and heart irradiated. Moreover, isocenter misalignments of 3 mm, with a negligible impact on the target’s dose coverage, maintain positive lung absolute dose differences *∆* V20 and *∆* V40 greater than 1.9% and 1.6%, respectively, and heart *∆* V25 difference not negligible in the posterior direction of 1.6%. In Fig. 2 are portrayed the 50 Gy and 20 Gy isodose lines of the reference and perturbed plan for different isocenter shifts for a representative patient. For 3 mm shifts the 50 Gy isodose line well encompasses the CTVth, while the 20 Gy isodose line includes a larger portion of heart and lung compared with the reference plan.

The higher the dose of the incidental radiation to the heart, the higher the probability of an event of cardiovascular complication or generally cardiotoxic effects [23–25]; this dose effect relationship forces the radiation oncologist to take in account that isocenter misplacement of few millimetres, may have consequence on the OARs when the reference doses were already close to the threshold of acceptability.

The results of this study were obtained using the patient data derived from the clinical practice of our department. The research does not have the value of a multi-institutional quality- assurance program, therefore the results cannot be generalized. Nevertheless, the findings obtained show that OARs dose deviations from the initial treatment conditions can arise for isocenter misplacement that slightly affect the target coverage.

Knowledge of dose coverage variation in chest wall tissue, adjacent lung, and heart are necessary to properly manage the accuracy of the treatment delivered along the treatment course. Isocenter positioning variations can be seen where the online match had failed on an individual day, although the neighbouring fractions were well matched. The use of custom margins in specific “critical” directions, for OARs, combined with the margins already in use by PTV, would help to limit the impact of possible positioning errors that may occur when setup support technologies (CBCT, image guidance, etc.) are not available or cannot be scheduled on a daily basis. Investigations performed with own equipment can highlight the impact on the plan dosimetry of isocenter positioning variations in specific directions; asymmetrical action levels could be considered for the daily verification.

## Conclusion

Inaccuracy in isocenter positioning for VMAT left-sided PMRT irradiation may impact the dosimetry of the CTVs and OARs to a different extent, depending on the directions and magnitude of the perturbation. The acquired information could be useful for planning strategies to guarantee the accuracy of the treatment delivered.

## Data Availability

The data are fully available without restriction in a public repository (Dryad). The reference treatment plans and corresponding perturbed plans were archived in the Informatic System of Sichuan Cancer Hospital.

## Abbreviations

VMAT: Volumetric modulated arc therapy
PMRT: Post mastectomy radiation therapy
CTV: Clinical target volume
DVH: Dose volume histogram
Dm: Mean dose
OARs: Organs at risk
CT: Computed tomography
CTVth: Thoracic wall clinical target volume
CTVsv: Supraclavicular clinical target volume
PTV: Planning target volume
CBCT: Cone beam computed tomography
